# Supplementary material for: Genetic Limitation and Conservation Implications in Tetracentron sinense: SNP-Based Analysis of Spatial Genetic Structure and Gene Flow
Source: Biology (Basel). 2025 Sep 8;14(9):1214. doi: 10.3390/biology14091214 (PMC12467406; doi:10.3390/biology14091214)
Supplement: Supplementary file 1 [file biology-14-01214-s001.zip › Supplementary data.pdf]

Supplementary Data

Table S1

population	Patches	Latitude	Longitude	Altitude	Number
BMXS	YA	27°37′25.69″-27°37′38.27″	99°21′22.52″-99°21′27.60″	2560-2762	32
	YB	27°37′51.36″-27°38′07.45″	99°21′14.27″-99°21′18.19″	2733-2882	32
	YC	27°38′02.94″-27°38′07.37″	99°21′05.72″-99°21′11.07″	2674-2828	33
GLGS	GA	26°22'57.92"-26°23'00.27"	108°11'46.55"-108°11'47.82"	1826-1840	36
	GB	26°22′44.56″-26°22′41.78″	108°12′42.80″-108°12′44.60″	1738-1772	36
	GC	26°22′59.83″-26°23′02.10″	108°11′51.45″-108°11′53.93″	1860-1885	33
MGFD	MA	28°46′37.41″-28°46′38.65″	103°08′33.42″-103°08′36.70″	2153-2206	46
	MB	28°46′07.77″-28°46′13.59″	103°08′30.86″-103°08′33.84″	2202-2255	37
	MC	28°46′20.58″-28°46′23.82″	103°08′36.07″-103°08′39.25″	2156-2283	46
SXFP	FA	33°38′51.32″-33°38′52.09″	107°49′07.19″-107°49′14.18″	1675-1756	13
	FB	33°38′53.34″-33°38′54.94″	107°49′17.02″-107°49′24.33″	1752-1797	16
	FC	33°38′54.36″-33°38′55.17″	107°49′36.06″-107°49′35.73″	1783-1840	18

Geographic information and number of individuals of 12 patches of *T.sinense*
